# Social Support and Optimism as Protective Factors for Mental Health among 7765 Healthcare Workers in Germany during the COVID-19 Pandemic: Results of the VOICE Study

**DOI:** 10.3390/ijerph18073827

**Published:** 2021-04-06

**Authors:** Caterina Schug, Eva Morawa, Franziska Geiser, Nina Hiebel, Petra Beschoner, Lucia Jerg-Bretzke, Christian Albus, Kerstin Weidner, Susann Steudte-Schmiedgen, Andrea Borho, Marietta Lieb, Yesim Erim

**Affiliations:** 1 Department of Psychosomatic Medicine and Psychotherapy, University Hospital of Erlangen, Friedrich-Alexander University Erlangen-Nürnberg (FAU), 91054 Erlangen, Germany; eva.morawa@uk-erlangen.de (E.M.); andrea.borho@uk-erlangen.de (A.B.); marietta.lieb@uk-erlangen.de (M.L.); yesim.erim@uk-erlangen.de (Y.E.); 2 Department of Psychosomatic Medicine and Psychotherapy, University Clinic of Bonn, 53127 Bonn, Germany; franziska.geiser@ukbonn.de (F.G.); nina.hiebel@ukbonn.de (N.H.); 3 Department of Psychosomatic Medicine and Psychotherapy, Ulm University Medical Center, 89081 Ulm, Germany; petra.beschoner@uniklinik-ulm.de (P.B.); lucia.bretzke@uni-ulm.de (L.J.-B.); 4 Department of Psychosomatics and Psychotherapy, University of Cologne, Medical Faculty and University Hospital, 50931 Cologne, Germany; christian.albus@uk-koeln.de; 5 Department of Psychotherapy and Psychosomatic Medicine, Faculty of Medicine, Technische Universität Dresden, 01307 Dresden, Germany; kerstin.weidner@uniklinikum-dresden.de (K.W.); susann.schmiedgen@tu-dresden.de (S.S.-S.)

**Keywords:** COVID-19, anxiety, depression, healthcare, mental health, resources, social support, optimism

## Abstract

Background: The COVID-19 pandemic is impacting mental health worldwide, particularly among healthcare workers (HCWs). Risk and protective factors for depression and generalized anxiety in healthcare workers need to be identified to protect their health and ability to work. Social support and optimism are known protective psychosocial resources, but have not been adequately studied in the context of the COVID-19 pandemic among healthcare workers in Germany. Methods: Within the first wave of the VOICE study (*n* = 7765), a longitudinal web-based survey study among healthcare workers in Germany, we assessed symptoms of depression (PHQ-2) and generalized anxiety (GAD-2), social support (ENRICHD Social Support Inventory; ESSI), and generalized optimism as well as sociodemographic, occupational, and COVID-19 related variables. Multiple linear regression analyses were conducted to examine associations between the constructs. Results: The analyses revealed that higher levels of social support and optimism were associated with lower levels of depression and generalized anxiety. They showed a higher association with depression and generalized anxiety than demographic or occupational risk factors such as female gender and direct contact with infected individuals. Conclusion: Psychosocial resources such as social support and optimism appear to contribute to successful coping with the COVID-19 pandemic and should be considered in future studies.

## 1. Introduction

The COVID-19 (Coronavirus Disease 2019) pandemic burdens societies worldwide. In this context, research has found increased mental distress in the general population of many countries, such as China [1], Italy [2], and Germany [1,3]. Several studies identified working in the healthcare sector as a risk factor for elevated mental burden during the COVID-19 pandemic (e.g., [1]). Some previous research reported mental health outcomes among healthcare workers (HCWs) during the COVID-19 pandemic in different countries: in Asian studies, prevalence rates of depression symptoms were documented between 5.3% (for at least moderate depression) and 50.4% [4]. In a sample of HCWs in Italy, 49.38% reported posttraumatic stress symptoms, 24.73% symptoms of depression, 80% symptoms of anxiety, 8.27% reported insomnia, and 21.90% high perceived stress [5]. For HCWs in Nepal, the symptoms of stress, anxiety, and depression were found to be 28.9%, 35.6%, and 17.0% respectively [6]. Morawa et al. [7] revealed prevalence rates of clinically significant levels of depression and anxiety symptoms to be 17.4% and 17.8% among physicians, and 21.6% and 19% among nurses working in hospitals in Germany.

Therefore, it is important to promote mental health in healthcare workers during the pandemic to support work ability in this highly relevant sector.

### 1.1. Risk Factors among Healthcare Workers

According to the conservation of resources theory [8], people need resources to maintain mental and physical well-being, and the potential or actual loss of resources represents the experience of distress. In the current study, we aim to examine resources as well as characteristics or conditions potentially threatening resources of HCW during the COVID-19 pandemic. We will refer to the first-named as “protective factors” and to the latter as “risk factors”.

#### 1.1.1. Gender and Direct Contact to Infected Patients

One of the most commonly reported risk factors of decreased mental health during the COVID-19 pandemic among HCWs was female gender. This finding was consistently reported by different systematic reviews and meta-analyses [9,10,11,12]. Working in direct contact with patients infected with COVID-19 was shown to be a correlate for adverse mental health outcomes among HCWs [12]. However, the opposite finding has also been shown as Li et al. [13] found that frontline nurses reported less distress than other nurses.

#### 1.1.2. Change of Department/Transfer to Another Work Department

As it was necessary to create new COVID-19 wards in hospitals or change work processes in other settings due to COVID-19, many HCWs had to change their work department. HCWs in China were even transferred to other cities or provinces [14]. It is well known that changes in the work environment are associated with increased stress, also among healthcare professionals (e.g., [15]). This leads to the assumption that HCWs who had to change their work department during the COVID-19 pandemic are more likely to show decreased mental health.

#### 1.1.3. Preexisting Illness

Another risk factor for mental burden might be having a preexisting illness. The Centers for Disease Control and Prevention in the United States reported that individuals in the following medical conditions are or might be at increased risk of developing a severe COVID-19 illness: cancer, chronic kidney disease, COPD (chronic obstructive pulmonary disease), heart conditions, immunocompromised state, overweight and obesity, sickle cell disease, diabetes mellitus, asthma, cerebrovascular disease, cystic fibrosis, hypertension or high blood pressure, neurologic conditions, liver disease, pulmonary fibrosis, and thalassemia [16]. The corresponding list of preexisting illnesses of the German Robert-Koch-Institute is mostly consistent with these findings [17]. Sayeed et al. [18] showed that the prevalence rates of stress, anxiety, and depression were significantly higher among individuals with a preexisting chronic illness than among healthy controls during the COVID-19 pandemic. Skoda et al. [19] demonstrated that participants with at least one preexisting somatic illness showed increased levels of COVID-19 related fear compared to healthy participants.

#### 1.1.4. Fear of Infection

Previous research also suggests that the fear of becoming infected or of infecting others is a common mental health problem [11]. Taylor et al. [20] found that the perceived dangerousness of COVID-19, including the fear of becoming infected and of infecting loved ones, was a central feature of the COVID stress syndrome. Its other features included worries about the socioeconomic costs of COVID-19, xenophobic fears, traumatic stress symptoms, and COVID-19-related compulsive checking and reassurance seeking. The syndrome was strongly associated with depression and anxiety [20].

### 1.2. Protective Factors

#### 1.2.1. Social Support

Social support refers to social interaction in which resources are received from others [21]. Social support helps people to manage uncertainty and increases the perception of personal control over one’s life [22].

The importance of human connection has been discussed for decades, whether it is called belongingness, social support [21], or the lack of loneliness [23]. In fact, missing social support or perceived loneliness is a significant risk factor for mortality, equal to or exceeding smoking, obesity, and not exercising, for those with chronic cardiac disease or for healthy individuals [23]. Concerning healthcare (HC) professionals, social support from both leaders and co-workers was found to be strongly connected to lower job strain and improved health outcomes [24,25,26,27]. Social support can reduce occupational stress [28] and perceptions of depersonalization [29]. It can prevent psychiatric symptoms and common mental disorders among HCWs [30]. Physicians described both senior and junior colleagues as an important source of social support [31]. Several studies demonstrated social support to be a protective factor for mental health during the COVID-19 pandemic for HCWs in Asia [32,33,34]. As cultures differ between continents and countries [35,36], the perception, manifestation, or protective role of social support might also. Therefore, we aim to examine social support in the context of HCWs in Germany during the COVID-19 pandemic.

#### 1.2.2. Optimism

Optimism has received attention in recent years due to the growing field of positive psychology [37]. Carver et al. [38] defined optimism as an individual difference variable that reflects the extent to which people hold generalized favorable expectancies for their future. Multiple studies showed that optimism is positively associated with psychological and physiological well-being [39], life satisfaction [40], and work satisfaction [41], and negatively associated with depression, suicide, and feelings of helplessness [40,42,43]. Although optimism is a relatively stable personality trait [44], it can change over time and be increased by interventions [45,46]. Optimism in HCWs has been shown to have a positive impact on the quality of life of nurses, to be associated with lower levels of burnout and with the personal accomplishment dimension of burnout [47,48]. Arslan et al. [49] found that optimism correlates negatively with COVID-19 related stress, somatization, anxiety, depression, and overall psychological problems in young Turkish adults. Concerning HCWs during the COVID-19 pandemic, optimism was shown to have a direct positive effect on work engagement and on organizational citizenship behavior [50], which are both favorable outcomes during a pandemic. Portuguese HCWs with higher levels in the hope/optimism dimension of the Spirituality Scale showed less COVID-19 related anxiety [51].

As optimism and social support have received very little attention in the research about protective factors and resources of HCWs during the COVID-19 pandemic in Germany, this study aims to clarify their role in promoting mental health alongside possible risk factors such as female gender, change of work department, direct contact with COVID-19 infected patients and a preexisting illness. Specifically, the current study uses the first wave data of the large prospective VOICE study (online survey of burden and psychosocial resources among medical staff during the COVID-19 pandemic) to address the following questions:

(1) Which group differences can be found between:
  - Men and women;
  - Those who changed the work department and those who did not;
  - Those who had direct contact with COVID-19 infected patients and those who did not;
  - Those with and without a preexisting illness concerning depression, generalized anxiety, fear of infection, social support, and optimism.
(2) Are the female gender, change of work department, direct contact with COVID-19 infected patients, and having preexisting illness risk factors with regard to depression and anxiety symptoms among HCWs in Germany during the COVID-19 pandemic?
(3) Are social support and optimism protective factors with regard to depression and anxiety symptoms among HCWs in Germany during the COVID-19 pandemic?

## 2. Materials and Methods

### 2.1. Data Collection

Between 20 April and 5 July 2020, a web-based survey was conducted as the first measurement of the prospective VOICE study that is part of the egePan Unimed (development, testing, and implementation of regionally adaptive care structures and processes for evidence-based pandemic management coordinated by the University Medical Center) project supported by the German Federal Ministry of Education and Research. The survey was developed by several longtime experienced experts from the field of psychosomatic medicine and psychotherapy. The authors tested and modified it multiple times for comprehensibility and feasibility. The psychosomatic departments of the university hospitals of Erlangen, Bonn, Ulm, Cologne, and Dresden shared the link via their intranets or mailing lists for the staff of their university hospitals (one reminder in Cologne) and several municipal hospitals. Various professional networks also promoted participation in the survey (e.g., Bavarian General Practitioners’ Association; Federal Working Group of the Social Pediatric Centers; Federal Association of Psychosomatics and Medical Psychotherapy; Federal Association of Occupational Medicine and an internet platform for physicians named Colliquio (one reminder)).

The 15 min survey was conducted in German via Unipark (www.unipark.com, accessed on 1 April 2021) and SoSci (www.soscisurvey.de, accessed on 1 April 2021), two academic online survey tools. The survey consisted of 77 items. Participants were asked to create a personal code to identify multiple participations of the same person and to enable data matching for a second survey. Inclusion criteria were a minimum age of 18 years, working in the healthcare sector, working place in Germany, and sufficient German language competency. The study was approved by the ethics committee of the University Hospital of Erlangen (reference number: 133_20 B). Informed consent was obtained from all individuals included in the study. Consent was given by actively ticking the consent checkbox and included consent to publication of non-identifying details. Due to the heterogenous recruitment strategy, the response rate for the total sample could not be measured. The response rates for the four participating university hospitals with the largest number of respondents were 10 (range: 8.1–13.3)% for physicians, 8.9 (range: 5.9–10.1)% for nurses, and 24.5 (range: 13.8–37.4)% for medical technical assistants. A plot of infected, deceased, and recovered COVID-19 cases in Germany over time displaying the context of the survey can be found in Morawa et al. [7].

### 2.2. Data Cleansing

Of 8236 cases with a filled in code, 123 were deleted because the identical codes indicated that the same person had answered the survey multiple times. The case with a higher number of completed items, and the newer one if the number was equal, remained in the dataset. A number of 29 cases were excluded because the participants indicated that they worked in another country other than Germany, 12 test participations were deleted. One case was deleted because of reporting being younger than 18 years. Of the 8071 remaining participants, 7765 completed all the sociodemographic and occupational items relevant to this study.

### 2.3. Measures

#### 2.3.1. Mental Health Variables

Depression symptoms were measured with the first two questions of the PHQ-D depression module (PHQ-2; Patient Health Questionnaire [52]). Participants indicated how often they experienced “feeling down, depressed, or hopeless” and “little interest or pleasure in doing things” over the last two weeks on a Likert scale from 0 = “not at all” to 3 = “nearly every day”. The theoretical range of the sum score is from 0 to 6. Higher scores reflect higher symptom severity. Scores ≥ 3 are considered probable clinically relevant cases of depression [53]. Cronbachs Alpha for the PHQ-2 was 0.75. The correlation between the two items was 0.603 (*p* < 0.001).

Anxiety symptoms were measured with the first two questions of the generalized anxiety module (GAD-2; [52]). Participants indicated how often they experienced “feeling nervous, anxious or on edge” and “not being able to stop or control worrying” over the last two weeks on a Likert scale from 0 = “not at all” to 3 = “nearly every day”. Theoretical range of the sum score is from 0 to 6. Higher scores reflect higher symptom severity. Scores ≥ 3 are considered probable clinically relevant cases of generalized anxiety [54]. Cronbachs Alpha for GAD-2 was 0.78. The correlation between the two items was 0.639 (*p* < 0.001).

#### 2.3.2. Psychosocial Resources

For assessing perceived emotional social support, we used the German version of the ENRICHD Social Support Inventory (ESSI) of Kendel et al. [55]. Its five items are answered on a five-point Likert scale from 1 = “never” to 5 = “always”. Example items are “if you need a conversation, is there someone who listens to you properly?” and “is there someone who shows you love and affection?”. The ESSI score is represented by the sum of all five item scores resulting in a range from 5 to 25. Higher scores reflect higher levels of perceived social support. Low social support is defined as a scale value of ≤18 and the answer of at least two items ≤3 [56]. Cronbach’s Alpha was 0.89.

Based on Kemper et al. [57] we assessed optimism with the item “how optimistic are you in general?” that was answered on a seven-point Likert-scale from 1 = “not optimistic at all” to 7 = “very optimistic”. Higher scores reflect higher levels of optimism.

#### 2.3.3. COVID-19 Related Variables

Fear of infection was measured with two self-generated items on a Likert scale from 0 = “strongly disagree” to 4 = “strongly agree” (with regard to the last two weeks): “I was afraid to become infected” and “I was afraid to infect relatives or my family.”

Furthermore, the following COVID-19 related variables were assessed by one item each: change of department during the pandemic (yes/no), having had direct contact at work with a COVID-19 infected patient proven by a test (yes/no), contact with contaminated material during work (yes/no), belonging to a risk group because of one’s age or a chronic illness (selection of none, one, or both options was possible), having been infected by the virus causing COVID-19 (yes/no/I do not know), degree of workload of the department (from 1 = “strongly below average” to 5 = “strongly above average”), and working in home office (yes, exclusively/yes, partly/no).

#### 2.3.4. Sociodemographic and Occupational Variables

The online questionnaire consisted of the following sociodemographic data: gender, age category (18–30/31–40/41–50/51–60/61–70/>70 years), living alone (or not), having children (or not), and migration background (present if the participant or at least one parent did not have the German citizenship by birth [58]). Occupational characteristics were the work setting, profession, years of professional experience, and working full-time or part-time.

The survey also included questionnaires measuring post-traumatic symptoms, working conditions, work–family conflict, and effort and reward imbalance at work. The results for these questionnaires are or will be analyzed in other publications. Morawa et al. [7] focused on the psychosocial burden and working conditions during the COVID-19 pandemic among physicians, nurses, and medical-technical assistants in hospitals.

### 2.4. Statistical Analysis

Data analyses were conducted with SPSS V. 24. Missing data were completed using the expectation-maximization algorithm (percentage of missing values: 7.4% for PHQ-2 and GAD-2, 2.8% for both fear of infection items, 7.7% for ESSI, 8.4% for optimism). Descriptive statistics (absolute and relative frequencies) were computed to profile the sociodemographic, occupational, and COVID-19 related characteristics of the sample. Group differences were tested with the t-test for independent samples. Effect sizes (Cohen’s d) are also reported (d ≥ 0.2 = small, d ≥ 0.5 = medium, and d ≥ 0.8 = large effect size; [59]). Multiple linear regression analyses were performed with the enter method to investigate the associations among sociodemographic, occupational, COVID-19 related variables, and psychosocial resource variables with the severity of depressive and anxiety symptoms. Independent variables with a β score ≥ 0.100 were considered as clinically relevant predictors following Cohen’s [59] classification for correlation coefficients. |β| is used to describe the absolute value of β without consideration of the signs. Alpha error level of *p* < 0.05 (two-tailed) was used for testing significance except for the case of alpha error correction according to Bonferroni (then explicitly reported in the text; 24 comparisons resulting in *p ≤* 0.05/24 ≤ 0.002). To examine relevant predictors for depression and anxiety symptoms among HCWs during the COVID-19 pandemic, multiple linear regression analyses were performed for the total sample and for the subgroups depending on gender, change of department, contact with infected patients, and preexisting illness. Included independent variables for the total sample were: gender, change of department, contact with infected patients, belonging to a risk group because of a preexisting illness, fear of becoming infected, fear of infecting family, social support, and optimism. Included control variables were: age (under/over 40 years old), migration background, hospital/other setting, working full-time/part-time, contact with patients in general, contact with contaminated material, and belonging to a risk group because of one’s age. In the subgroup analyses, all listed variables but the grouping variable were included as independent variables.

## 3. Results

### 3.1. Sample Characteristics

Table 1 and Table 2 show sociodemographic, occupational, and COVID-19 related characteristics of the sample. The sample of 7765 HCW consisted of 76.3% women. The most frequent age category was 51–60 years (28.4%). The participants had the following professions: 1909 physicians (24.6%), 1335 nurses (17.2%), 1770 medical-technical staff (22.8%), 481 pedagogues and educators in the healthcare sector (6.2%), 450 administration staff (with and without direct patient contact) (5.8%), 402 psychologists (5.2%), and 1418 (18.3%) of other professions. A proportion of 38.9% had direct contact with COVID-19 infected patients at work and 19.6% reported to be in a health condition that heightens the risk of a severe COVID-19 course. A proportion of 13.6% had changed their work department during the COVID-19 pandemic. Only 1% had been infected by the novel virus.

### 3.2. Levels of Depression, Generalized Anxiety, and Fear of Infection

In Table 3, depression and anxiety scores among the sample of HCWs are presented. The proportion of probable cases of clinical depression (scores ≥ 3) was 19% in the total sample. The prevalence rate of a probable depression was highest among those with a preexisting illness (25.1%) and lowest in those without a preexisting illness (17.6%). The prevalence rate of probable cases of a clinical generalized anxiety (scores ≥ 3) was also 19% in the total sample. This prevalence rate was highest among those with a preexisting illness (27.1%) and lowest in men (16.5%).

Table 4 shows scores of fear of becoming infected and fear of infecting relatives. The mean score of fear of becoming infected was 1.66 (SD = 1.22) in the total sample and ranged from 1.55 (SD = 1.18; those without a preexisting illness) to 2.09 (SD = 1.28; those with a preexisting illness) in the subsamples. The mean score of fear of infecting relatives was 2.28 (SD = 1.32) in the total sample and ranged from 2.08 (SD = 1.34; men) to 2.53 (SD = 1.30; those with direct contact with COVID-19 infected patients).

### 3.3. Levels of Social Support and Optimism

Table 5 presents scores for social support and general optimism among the sample of healthcare workers. Of the total sample, 21.9% had an ESSI value ≤18 indicating a low level of social support. Low social support was most frequent in the group with a preexisting illness (29.2%) and least frequent in those without a preexisting illness (20.2%).

### 3.4. Group Differences in Depression, Generalized Anxiety, Fear of Infection, Social Support, and Optimism

#### 3.4.1. Differences Depending on Gender

Women showed significantly higher depression symptoms (*p =* 0.001; d = 0.09, negligible effect) and higher symptoms of generalized anxiety (*p ≤* 0.001; d = 0.17, very small to small effect) than men. Female HCWs feared becoming infected (*p ≤* 0.001; d = 0.10, very small effect size) and infecting relatives (*p ≤* 0.001; d = 0.20, small effect size) significantly more than male HCWs. Concerning psychosocial resources, female HCWs perceived higher levels of emotional social support (*p ≤* 0.001; d = 0.11, very small effect). There were no significant gender differences in general optimism.

#### 3.4.2. Differences Depending on Change of Work Department

Department changers showed elevated symptoms of depression (*p ≤* 0.001; d = 0.16, very small to small effect size) and generalized anxiety (*p ≤* 0.001; d = 0.13, very small to small effect) and feared infecting their relatives more than non-department changers (*p =* 0.002; d = 0.10, very small effect).

#### 3.4.3. Differences Depending on Direct Contact with COVID-19-Infected Patients

The HCWs who had direct contact with COVID-19-infected patients (HCW-DC+) did not differ significantly from those who did not have direct contact (HCW-DC–) in depression or anxiety symptoms nor in their psychosocial resources. HCW-DC+ reported more fear of becoming infected (*p ≤* 0.001; d = 0.25, small effect) and of infecting their family (*p ≤* 0.001; d = 0.32, small effect) than HCW-DC–.

#### 3.4.4. Differences Depending on Belonging to a Risk Group Because of a Preexisting Illness

The participants who indicated belonging to a risk-group for a severe course of COVID-19 because of a preexisting illness (HCW-PE+) showed higher depression symptoms (*p ≤* 0.001; d = 0.21, small effect) and higher anxiety symptoms (*p ≤* 0.001; d = 0.27, small effect size) compared to those not indicating this risk characteristic (HCW-PE–). HCWs-PE+ feared becoming infected (*p ≤* 0.001; d = 0.43, small to medium effect) and infecting their relatives (*p ≤* 0.001; d = 0.18, very small to small effect) more, perceived less emotional social support (*p ≤* 0.001; d = 0.23, small effect), and described themselves as less optimistic (*p ≤* 0.001; d = 0.13, very small to small effect) than HCWs-PE–.

### 3.5. Risk Factors and Protective Factors for Depression and Anxiety Symptoms

The strongest independent variable associated with depression symptoms in the regression analysis for the total sample was social support, with higher levels of social support being associated with lower levels of depression symptoms (Table 6). For PHQ-2, the adjusted R^2^ value was 18.0% (moderate level of explained variance [50]). Fear of becoming infected (β = 0.100), fear of infecting relatives (β = 0.107), social support (β = −0.230), and optimism (β = −0.204) were statistically significant and clinically relevant independent variables. Fear of infection was associated with higher depression levels whereas social support and optimism were associated with lower depression levels. Gender, change of department, contact with infected patients, and belonging to a risk-group because of a preexisting illness were statistically significant independent variables, but their clinical relevance was negligible (|β| < 0.042). Age (under/above 40 years) and working full-time/part-time were also statistically significant, but of negligible clinical relevance (|β| < 0.057).

The strongest independent variable related to symptoms of generalized anxiety was social support, higher levels of social support being associated with lower levels of generalized anxiety. For GAD-2, the adjusted R^2^ value was 21.2% (moderate to high level of explained variance [50]). Fear of becoming infected (β = 0.187), fear of infecting relatives (β = 0.108), social support (β = −0.214), and optimism (β = −0.203) were statistically significant and clinically relevant independent variables. Fear of infection was associated with higher levels of generalized anxiety symptoms whereas social support and optimism were associated with lower anxiety levels. Female gender, change of department, and belonging to a risk group because of a preexisting illness were statistically significant independent variables, but their clinical relevance was negligible (|β| < 0.064). Direct contact with COVID-19 infected patients was not a significant independent variable in this model. Work setting was a significant independent variable but also of negligible clinical relevance (β = 0.045).

### 3.6. Risk and Protective Factors in Subgroup Analyses

Social support and optimism were significant and clinically relevant independent variables associated with lower scores of depressive and anxiety symptoms in the total sample as well as in all subgroups. Specifically, higher levels of social support were broadly associated with lower depressive and anxiety symptoms in the total sample as well as in most subgroups. Optimism was the variable most strongly associated with decreased depression and anxiety symptoms among men and with decreased anxiety symptoms in HCWs-DC–. (Supplementary Materials Tables S1–S9).

The adjusted R^2^ values of the regression models for depression and anxiety performed for the eight subgroups ranged from 14.6% (prediction of depressive symptoms for department changers, moderate explanation of variance) to 24.0% (prediction of generalized anxiety symptoms in men, strong explanation of variance).

## 4. Discussion

The present study aimed to identify risk and protective factors for symptoms of depression and generalized anxiety in healthcare workers during the COVID-19 pandemic under special consideration of the psychosocial resources social support and optimism. It provides an application of protective and risk factors to the burden of HCWs in Germany during the COVID-19 pandemic.

Whereas only 1% of the staff had been infected by COVID-19 at the study time, every fifth HCW showed symptoms of depression or anxiety that are considered as probably clinically relevant on the basis of established cut-off values ([53,54]). In line with Taylor et al. [20], this underlines that the psychosocial footprint of the COVID-19 pandemic is likely to be broader than its purely medical footprint, also for professionals in the healthcare sector. The damage caused by the pandemic seems to be more pronounced in depression and anxiety symptoms than in physical health within the sample.

Before the COVID-19 pandemic, HCWs seemed to be more mentally burdened than other professions, for example in the UK [60,61,62] and Germany [63]. In the UK, the prevalence of common mental health disorders was higher in health and related occupations (19% (95% CI 13–25%) than in all occupations (13%) and was 19% (6–33%) in nursing auxiliaries [61,62]. Although there is no data available for direct comparison of prevalence rates between HCWs and other professions in Germany, Beschoner et al. [63] reported prevalence rates among physicians in Germany between 4% and 20% for burnout and between 6% and 13% for depression, which are slightly higher than the prevalence rates of depression (8.1%) and of burnout (4.2%) found in the German general population [64]. However, during the current pandemic, HCWs in Germany do not seem to be more impacted in terms of depression and anxiety symptoms than the general population as 25% clinically relevant GAD-2 and PHQ-2 scores were documented for the general population [65]. The result could be due to the earlier survey time among the general population or that HCWs might show better coping skills for pandemic distress than other professions.

In line with previous research, female gender and change of work department were identified as significant variables of risk for depression and anxiety, but with relatively low effect sizes leading to the assumption that other variables might be more important in differentiating risk groups for depression and anxiety. This finding matches the results of Buselli et al. [66] that gender was not a significant predictor for depression nor anxiety among HCWs during the COVID-19 pandemic in Italy. In both studies, the result could be impacted by the fact that a clear majority of the sample were women. The present study did not find empirical evidence that direct contact with COVID-19 patients constitutes a general variable of risk for depression and anxiety. In Buselli et al. [66], frontline activity was not a significant risk factor for depression, but for anxiety. This is in line with our results. However, for one subgroup (department changers), frontline activity even seemed to potentially have a protective value against anxiety, which is contradicting the finding of Buselli et al. [66], but confirming the findings of Li et al. [13]. There were no group differences in social support depending on the change of department although those who had to change the department need to integrate themselves into a new social work context. Possibly, the change in social support is not yet detectable shortly after the change of department. Furthermore, social support from the private context might buffer a lack of social support in the workplace.

The presence of a preexisting illness seems to be a variable of risk for depression and anxiety in comparison to the lack of a preexisting illness, albeit with a small effect size. This finding needs to be interpreted considering that the presence of a chronic illness increases depression likelihood also in non-pandemic times [67]. Nevertheless, this burden persists and should also be considered during a pandemic. Our finding about preexisting illnesses is in line with Sayeed et al. [18]. However, when resources and fear of infection are taken into account, a preexisting illness seems to have little significance as an independent variable for depression and anxiety symptoms.

As expected and consistent with previous research [21,51], social support and general optimism were associated with less depression and less general anxiety symptoms. They were the independent variables most associated with lower levels of depression and anxiety, underpinning their potential protective role for mental health in all subgroups of HCWs. As the study design is cross-sectional, it might be possible that higher levels of social support and optimism cause lower levels of depression and anxiety, but also that decreased levels of depression and anxiety may result in a more positive perception of psychosocial resources, or that both are true.

The variables most associated with elevated symptoms of depression and anxiety were fear of infection and fear of infecting relatives, which is in line with previous research (e.g., [20]). While the fear of becoming infected was clinically relevantly related to anxiety symptoms in all groups, its role for depression symptoms seemed to be more relevant (regarding effect sizes) within the groups with a higher fear of becoming infected (women, department changers, and those with a preexisting illness).

Although the expected risk characteristics such as female gender, change of work department, direct contact with COVID-19 patients, and the presence of a preexisting illness showed relatively small or negligible effect sizes as correlates of depression and anxiety symptoms, they were nonetheless linked to a higher fear of becoming infected and fear of infecting relatives. Due to the correlation between these fears and increased symptoms of depression and anxiety, as well as the fact that the data was collected during a relatively early phase of the COVID-19 pandemic, there is reason to believe that the full effects of these particular characteristics on mental health might only be detectable during later stages in the pandemic. This was similarly the case during the severe acute respiratory syndrome (SARS) epidemic, in which the delayed effects of depression were frequently observed [68,69].

### 4.1. Limitations

One limitation of the study was that all data were self-reported, and therefore lack the possibility of objective verification. However, the anonymous self-reported character was necessary to protect identities for ethical purposes. The design only permitted a self-selected sample within the addressed HCWs so that it might be possible that either especially burdened or especially resourceful HCWs participated. Additionally, depression and anxiety symptoms were surveyed with only two items each and optimism with one item, possibly reducing criterion validity. These limits were nonetheless necessary for this study in order to increase the economy and ease of use for a target professional population that is already challenged by the pandemic. We did not measure isolation due to unprotected exposure to cases tested positive for SARS-CoV-2; therefore, we were unable to take this variable into account. Another limitation was the cross-sectional design of this analysis. The survey is planned with a longitudinal design but in order to share our results with the scientific community quickly, we conducted a cross-sectional analysis. Data collection for longitudinal analyses is in progress. Lastly, the heterogeneous variety of professions in the sample creates a risk of undetected intervening variables due to the inclusion of diverse professions. On the other hand, the heterogeneity of professions and the sample size support the generalizability of the findings considering the heterogeneity of healthcare workers essential for coping with the current pandemic.

### 4.2. Contribution

The study contributes to the research field of mental health, psychosocial resources, and positive psychology in times of a worldwide pandemic as it provides evidence for social support and general optimism as important protective variables for depression and anxiety symptoms in HCWs in Germany during the COVID-19 pandemic. Whereas previous research has often focused on demographic and occupational characteristics when examining mental health during the COVID-19 pandemic, this study supposes that the significance of gender, change of work department, and direct contact with COVID-19 infected patients is less substantial with regard to depression and anxiety than fear of infection and individual psychosocial resources. Nevertheless, female gender, change of work department, and direct contact with COVID-19 infected patients seem to be variables of risk for experiencing fear of infection, which is positively associated with depression and anxiety symptoms. When focusing on possible risk groups among HCWs, those with a preexisting illness should not be forgotten because they are at risk in multiple perspectives: in a medical perspective when becoming infected with COVID-19 and in psychosocial perspectives as they are more likely to show depression and anxiety symptoms while perceiving fewer resources such as social support and optimism.

### 4.3. Implications

The findings of the study suggest that maintaining or enhancing social support and optimism in the workplace might be beneficial for protecting mental health and work ability of HCW during the COVID-19 pandemic. Social support within the work team can be promoted for example by team-building measures, adequate leadership, and by providing sufficient opportunities for social support in the private context (leisure time). It should be noted that online means of maintaining social connection gain importance during a pandemic, including for example telemedicine and informal support groups [70]. Optimism could be considered in psychoeducational employee trainings in the workplace. Those responsible in the workplace and politics should take into account that especially those who change their work department, have direct contact with COVID-19 patients and those with a preexisting illness are more likely to fear infection of the self and the loved ones, possibly impacting their mental health. The workplace could, therefore, offer psychosocial care such as hotlines or low threshold opportunities in the ward or work department where possible. Further research should thus address the question of whether and how social support and optimism can effectively contribute to mental health support in the workplace during pandemics.

## 5. Conclusions

Psychosocial resources such as social support and optimism seem to play a role in the mental health of HCWs in Germany during the COVID-19 pandemic. Strengthening these resources may be beneficial for protecting their mental health and work ability.

Taking into account psychosocial resources and fear of infection help to understand mental health in medical staff during the COVID-19 pandemic better than focusing on demographic or occupational characteristics only.

Future prospective studies should address social support, optimism, and other psychosocial resources as influencing factors for mental health in healthcare professionals during pandemics.

## Figures and Tables

**Table 1:** Socio-demographic and occupational characteristics of the study sample.

	Total Sample *n* = 7765
Gender, n (%)	
Women	5921 (76.3)
Men	1819 (23.4)
Divers	25 (0.3)
Age, years, n (%)	
18–30	1483 (19.1)
31–40	1753 (22.6)
41–50	1778 (22.9)
51–60	2208 (28.4)
61–70	511 (6.6)
>70	32 (0.4)
Living alone, n (%)	
Yes	1693 (21.8)
No	6072 (78.2)
Children, n (%)	
Yes, in the household	3062 (39.4)
Yes, but not in the household	1256 (16.2)
No	3447 (44.4)
Migration background, n (%)	
Yes	846 (10.9)
No	6919 (89.1)
Professional experience in patient care, n (%)	
<3 years	768 (9.9)
3–6 years	790 (10.2)
>6 years	4896 (63.1)
Not in direct patient care	1311 (16.9)
Employment, n (%)	
Full-time	4832 (62.2)
Part-time	2933 (37.8)
Work setting, n (%)	
University hospital	3340 (43.0)
Non-university hospital	1635 (21.1)
Private practice	741 (9.5)
Medical care center	238 (3.1)
Social pediatric/ Early intervention facility	1264 (16.3)
Other (e.g., occupational health facility, ambulant care, laboratory)	547 (7.0)

**Table 2:** COVID-19 related characteristics of the study sample.

	Total Sample *n* = 7765
Contact with infected patients, n (%)	
Yes	3024 (38.9)
No	4741 (61.1)
Contact with contaminated material, n (%)	
Yes	3011 (38.8)
No	4754 (61.2)
Risk group age, n (%)	
Yes	6645 (85.6)
No	1120 (14.4)
Risk group preexisting illness, n (%)	
Yes	1520 (19.6)
No	6245 (80.4)
No risk group, n (%)	
Yes	5524 (71.1)
No	2241 (28.9)
Infection with SARS-CoV-2 virus, n (%)	
Yes	77 (1.0)
No	4190 (54.0)
I don’t know	3498 (45.0)
Workload of the department, n (%)	
Strongly below average	1210 (15.6)
Slightly below average	2350 (30.3)
Average	2258 (29.1)
Slightly above average	1181 (15.2)
Strongly above average	766 (9.9)
Change of department, n (%)	
Yes	1059 (13.6)
No	6706 (86.4)
Home office, n (%)	
Yes, exclusively	215 (2.8)
Yes, partly	1504 (19.4)
No	6046 (77.9)

**Table 3:** Depression and anxiety symptoms among the sample of healthcare workers.

			PHQ-2 Items	GAD-2 Items
		*n*	M (SD); (Score ≥ 3 in %)	M (SD); (Score ≥ 3 in %)
Total sample		7765	1.63 (1.39); (19.0)	1.55 (1.45); (19.0)
Gender	male	1819	1.53 (1.39); (18.6)	1.36 (1.40); (16.5)
	female	5921	1.66 (1.39); (19.2)	1.61 (1.46); (19.7)
			*p ≤* 0.001; d = 0.09	*p ≤* 0.001; d = 0.17
Change of work department	yes	1059	1.83 (1.50); (23.5)	1.72 (1.52); (23.3)
	no	6706	1.59 (1.37); (18.3)	1.52 (1.44); (18.3)
			*p ≤* 0.001; d = 0.16	*p ≤* 0.001; d = 0.13
Direct contact with COVID-19 patients	yes	3024	1.65 (1.40); (19.9)	1.57 (1.48); (19.9)
	no	4741	1.61 (1.38); (18.5)	1.53 (1.43); (18.4)
			n.s.	n.s.
Risk group because of preexisting illness	yes	1520	1.87 (1.49); (25.1)	1.89 (1.58); (27.1)
	no	6245	1.57 (1.36); (17.6)	1.47 (1.41); (17.0)
			*p ≤* 0.001; d = 0.21	*p ≤* 0.001; d = 0.27

*p* and d values of t-tests for the mean scores are reported underneath the two compared groups if *p* < 0.05. n.s. = not significant. The significant results remain significant after alpha error correction according to Bonferroni (*p ≤* 0.002). PHQ-2 = short form of the depression module of the Patient Health Questionnaire; GAD-2 = short form of the generalized anxiety module of the Patient Health Questionnaire; COVID-19 = Coronavirus Disease 2019.

**Table 4:** Fear of infection among the sample of healthcare workers.

			Fear of Becoming Infected	Fear of Infecting Relatives
		*n*	M (SD)	M (SD)
Total sample		7765	1.66 (1.22)	2.28 (1.32)
Gender	male	1819	1.56 (1.23)	2.08 (1.34)
	female	5921	1.69 (1.22)	2.34 (1.31)
			*p ≤* 0.001; d = 0.10	*p ≤* 0.001; d = 0.20
Change of work department	yes	1059	1.67 (1.24)	2.40 (1.32)
	no	6706	1.66 (1.22)	2.26 (1.32)
			n.s.	*p =* 0.002; d = 0.10
Direct contact with COVID-19 patients	yes	3024	1.85 (1.27)	2.53 (1.30)
	no	4741	1.54 (1.17)	2.12 (1.32)
			*p ≤* 0.001; d = 0.25	*p ≤* 0.001; d = 0.32
Risk group because of preexisting illness	yes	1520	2.09 (1.28)	2.47 (1.33)
	no	6245	1.55 (1.18)	2.23 (1.32)
			*p ≤* 0.001; d = 0.43	*p ≤* 0.001; d = 0.18

*p* and d values of t-tests for the mean scores are reported underneath the two compared groups if *p* < 0.05. n.s. = not significant. The significant results remain significant after alpha error correction according to Bonferroni (*p ≤* 0.002).

**Table 5:** Social support and general optimism among the sample of healthcare workers.

			Social Support (ESSI)	Optimism
		*n*	M (SD); (Score ≤ 18 in %)	M (SD)
Total sample		7765	20.74 (3.84); (21.9)	5.17 (1.29)
Gender	male	1819	20.43 (3.96); (24.9)	5.15 (1.31)
	female	5921	20.84 (3.78); (21.0)	5.18 (1.29)
			*p ≤* 0.001; d = 0.11	n.s.
Change of work department	yes	1059	20.53 (3.94); (24.9)	5.12 (1.28)
	no	6706	20.77 (3.82); (21.5)	5.18 (1.30)
			n.s.	n.s.
Direct contact with COVID-19 patients	yes	3024	20.66 (3.94); (23.2)	5.17 (1.32)
	no	4741	20.79 (3.77); (21.2)	5.17 (1.28)
			n.s.	n.s.
Risk group because of preexisting illness	yes	1520	20.00 (4.16); (29.2)	5.04 (1.33)
	no	6245	20.92 (3.74); (20.2)	5.20 (1.28)
			*p ≤* 0.001; d = 0.23	*p ≤* 0.001; d = 0.13

*p* and d values of *t*-tests for the mean scores are reported underneath the two compared groups if *p* < 0.05. n.s. = not significant. The significant results remain significant after alpha error correction according to Bonferroni (*p ≤* 0.002).

**Table 6:** Linear regression analyses for the severity of depressive (PHQ-2) and generalized anxiety symptoms (GAD-2) for the total sample of healthcare workers.

	PHQ-2 R^2^_adj_ = 18.0%							GAD-2 R^2^_adj_ = 21.2%						
	Regression Coefficient	95% CI: Minimum	95% CI: Maximum	SE	β	T	*p*-Value	Regression Coefficient	95% CI: Minimum	95% CI: Maximum	SE	β	T	*p*-Value
Constant	4.157	3.949	4.366	0.106		39.123	**≤0.001**	3.623	3.409	3.836	0.109		33.298	**≤0.001**
Gender (male vs. female)	0.139	0.070	0.208	0.035	0.042	3.927	**≤0.001**	0.220	0.149	0.291	0.036	0.064	6.086	**≤0.001**
Age (under vs. over 40)	−0.161	−0.225	−0.098	0.033	−0.057	−4.957	**≤0.001**	0.052	−0.014	0.117	0.033	0.018	1.545	0.122
Migration background (no vs. yes)	0.007	−0.084	0.098	0.046	0.002	0.150	0.881	0.030	−0.063	0.123	0.047	0.006	0.624	0.533
Work Setting (Hospital vs. Other)	−0.012	−0.072	0.049	0.031	−0.004	−0.375	0.708	0.135	0.073	0.197	0.032	0.045	4.244	**≤0.001**
Employment (Fulltime vs. Part time)	−0.089	−0.150	−0.028	0.031	−0.031	−2.866	**0.004**	0.004	−0.059	0.066	0.032	0.001	0.115	0.908
Contact with patients (yes vs. no)	0.075	−0.003	0.153	0.040	0.020	1.881	0.060	0.030	−0.050	0.110	0.041	0.008	0.739	0.460
Change of department (yes vs. no)	−0.181	−0.263	−0.099	0.042	−0.045	−4.308	**≤0.001**	−0.160	−0.244	−0.076	0.043	−0.038	−3.722	**≤0.001**
Direct contact with infected patients (yes vs. no)	0.089	0.011	0.168	0.040	0.031	2.227	**0.026**	0.075	−0.005	0.156	0.041	0.025	1.840	0.066
Contact with contamined material (yes vs. no)	−0.037	−0.113	0.039	0.039	−0.013	−0.961	0.337	−0.066	−0.144	0.012	0.040	−0.022	−1.664	0.096
Risk group age (no vs. yes)	0.012	−0.074	0.098	0.044	0.003	0.269	0.788	−0.051	−0.139	0.037	0.045	−0.012	−1.132	0.258
Risk group preexisting illness (no vs. yes)	0.131	0.057	0.204	0.037	0.037	3.500	**≤0.001**	0.161	0.086	0.236	0.038	0.044	4.213	**≤0.001**
Fear of becoming infected	0.113	0.083	0.144	0.015	0.100	7.376	**≤0.001**	0.222	0.191	0.253	0.016	0.187	14.092	**≤0.001**
Fear of infecting family	0.113	0.085	0.140	0.014	0.107	7.949	**≤0.001**	0.118	0.090	0.147	0.014	0.108	8.154	**≤0.001**
Social Support (ESSI)	−0.083	−0.091	−0.076	0.004	−0.230	−21.299	**≤0.001**	−0.081	−0.089	−0.073	0.004	−0.214	−20.201	**≤0.001**
Optimism	−0.220	−0.242	−0.197	0.012	−0.204	−18.992	**≤0.001**	−0.228	−0.251	−0.205	0.012	−0.203	−19.275	**≤0.001**

R^2^_adj_ = adjusted R^2^ (explained variance); SE = standard error; (manifestation coded by 0 vs. manifestation coded by 1); *n* = 7740. Significant p-values are marked in bold.

## Data Availability

The data presented in this study are available on request from the corresponding author. The data are not publicly available as the approval of the Ethics Commitee does not include this type of data publication.

## Supplementary Materials

The following are available online at https://www.mdpi.com/article/10.3390/ijerph18073827/s1, Table S1: Linear regression analysis for severity of depressive (PHQ-2) symptoms for the subgroups depending on gender, Table S2: Linear regression analysis for severity of generalized anxiety (GAD-2) symptoms for the subgroups depending on gender, Table S3: Linear regression analysis for severity of depressive (PHQ-2) symptoms for the subgroups depending on change of department, Table S4: Linear regression analysis for severity of generalized anxiety (GAD-2) symptoms for the subgroups depending on change of department, Table S5: Linear regression analysis for severity of depressive (PHQ-2) symptoms for the subgroups depending on direct contact with Covid-19 infected patients, Table S6: Linear regression analysis for severity of generalized anxiety (GAD-2) symptoms for the subgroups depending on direct contact with COVID-19 infected patients, Table S7: Linear regression analysis for severity of depressive (PHQ-2) symptoms for the subgroups depending on the presence of a preexisting illness, Table S8: Linear regression analysis for severity of generalized anxiety (GAD-2) symptoms for the subgroups depending on the presence of a preexisting, Table S9: Independent variables (IV) for severity of depressive and generalized anxiety symptoms in the subgroups men, women, department changers, non-department changers, those with/without direct contact to COVID-19 patients and those with/without a preexisting illness.
