# Supplementary material for: Social Support and Optimism as Protective Factors for Mental Health among 7765 Healthcare Workers in Germany during the COVID-19 Pandemic: Results of the VOICE Study
Source: Int J Environ Res Public Health. 2021 Apr 6;18(7):3827. doi: 10.3390/ijerph18073827 (PMC8038794; doi:10.3390/ijerph18073827)
Supplement: Supplementary file 1 [file ijerph-18-03827-s001.pdf]

Supplement 1 Linear regression analysis for severity of depressive (PHQ-2) symptoms for the subgroups depending on gender

PHQ2	Male R^2_{adj} = 21.4 %							Female R^2_{adj} = 16.8 %						
	Regression coefficient	95% CI: minimum	95% CI: maximum	SE	β	T	P value	Regression coefficient	95% CI: minimum	95% CI: maximum	SE	β	T	P value
Konstante	4.185	3.788	4.581	0.202		20.701	≤.000	4.292	4.050	4.535	0.124		34.640	≤ .001
Age (under/over 40)	-0.127	-0.259	0.006	0.068	-0.044	-1.869	.062	-0.171	-0.244	-0.097	0.037	-0.061	-4.566	≤ .001
Migration background	0.097	-0.095	0.288	0.098	0.021	0.992	.321	-0.015	-0.118	0.089	0.053	-0.003	-0.281	.779
Hospital/ Other setting	0.084	-0.042	0.211	0.064	0.028	1.313	.189	-0.035	-0.105	0.035	0.036	-0.012	-0.974	.330
Fulltime/ Part time	-0.061	-0.220	0.098	0.081	-0.016	-0.755	.451	-0.091	-0.157	-0.025	0.034	-0.033	-2.688	.007
Contact with patients	0.050	-0.118	0.219	0.086	0.013	0.586	.558	0.077	-0.011	0.166	0.045	0.021	1.712	.087
Change of department	-0.299	-0.461	-0.138	0.082	-0.077	-3.631	≤ .001	-0.142	-0.237	-0.046	0.049	-0.035	-2.898	.004
Contact with infected patients	0.108	-0.060	0.277	0.086	0.039	1.261	.207	0.085	-0.004	0.175	0.046	0.030	1.872	.061
Contact with contamined material	-0.019	-0.185	0.147	0.085	-0.007	-0.224	.823	-0.047	-0.133	0.039	0.044	-0.016	-1.066	.287
Risk group age	0.042	-0.117	0.201	0.081	0.012	0.521	.603	-0.007	-0.110	0.096	0.052	-0.002	-0.136	.892
Risk group preexisting illness	0.164	0.014	0.313	0.076	0.047	2.151	.032	0.121	0.037	0.205	0.043	0.035	2.820	.005
Fear of becoming infected	0.099	0.035	0.162	0.032	0.087	3.051	.002	0.116	0.082	0.151	0.018	0.102	6.637	≤ .001
Fear of infecting family	0.150	0.091	0.208	0.030	0.143	4.992	≤ .001	0.102	0.070	0.134	0.016	0.097	6.335	≤ .001
Social Support (ESSI)	-0.080	-0.096	-0.065	0.008	-0.229	-10.415	≤ .001	-0.085	-0.093	-0.076	0.005	-0.230	-18.521	≤ .001
Optimism	-0.246	-0.292	-0.200	0.023	-0.230	-10.459	≤ .001	-0.211	-0.237	-0.185	0.013	-0.196	-15.858	≤ .001

R^2_{adj} = adjusted R^2 (explained variance); SE = standard error; (manifestation coded by 0 vs. manifestation coded by 1); N = 7740

Supplement 2 Linear regression analysis for severity of generalized anxiety (GAD-2) symptoms for the subgroups depending on gender

GAD2	Male<br>R2adj = 24.0 %							Female<br>R2adj = 19.9 %						
	Regression coefficient	95% CI: minimum	95% CI: maximum	SE	β	T	P value	Regression coefficient	95% CI: minimum	95% CI: maximum	SE	β	T	P value
Konstante	3.452	3.061	3.844	0.200		17.282	≤ .001	3.905	3.654	4.156	0.128		30.469	≤ .001
Age (under/over 40)	0.055	-0.076	0.186	0.067	0.019	0.823	.410	0.046	-0.030	0.122	0.039	0.016	1.193	.233
Migration background	0.110	-0.079	0.299	0.096	0.024	1.139	.255	0.011	-0.097	0.118	0.055	0.002	0.193	.847
Hospital/ Other setting	0.167	0.043	0.292	0.064	0.056	2.630	.009	0.130	0.057	0.202	0.037	0.043	3.515	≤ .001
Fulltime/ Part time	-0.033	-0.190	0.124	0.080	-0.008	-0.410	.682	0.011	-0.057	0.080	0.035	0.004	0.322	.747
Contact with patients	-0.010	-0.177	0.157	0.085	-0.002	-0.113	.910	0.037	-0.055	0.128	0.047	0.010	0.784	.433
Change of department	-0.239	-0.399	-0.080	0.081	-0.061	-2.938	.003	-0.133	-0.232	-0.034	0.051	-0.031	-2.624	.009
Contact with infected patients	0.048	-0.119	0.214	0.085	0.017	0.562	.574	0.077	-0.016	0.169	0.047	0.025	1.624	.105
Contact with contamined material	0.016	-0.148	0.180	0.084	0.006	0.189	.850	-0.087	-0.176	0.002	0.045	-0.029	-1.922	.055
Risk group age	-0.083	-0.240	0.074	0.080	-0.023	-1.036	.300	-0.043	-0.149	0.064	0.054	-0.010	-0.784	.433
Risk group preexisting illness	0.233	0.085	0.380	0.075	0.066	3.099	.002	0.139	0.052	0.226	0.044	0.038	3.141	.002
Fear of becoming infected	0.231	0.168	0.294	0.032	0.203	7.231	≤ .001	0.218	0.183	0.254	0.018	0.182	12.051	≤ .001
Fear of infecting family	0.127	0.069	0.185	0.030	0.121	4.284	≤ .001	0.115	0.082	0.148	0.017	0.104	6.908	≤ .001
Social Support (ESSI)	-0.070	-0.085	-0.055	0.008	-0.198	-9.181	≤ .001	-0.085	-0.094	-0.075	0.005	-0.219	-17.912	≤ .001
Optimism	-0.241	-0.286	-0.195	0.023	-0.225	-10.372	≤ .001	-0.224	-0.251	-0.197	0.014	-0.198	-16.265	≤ .001

R^2_{adj} = adjusted

R^2 (explained variance); SE = standard error; (manifestation coded by 0 vs. manifestation coded by 1); N = 7740

Supplement 3 Linear regression analysis for severity of depressive (PHQ-2) symptoms for the subgroups depending on change of department

PHQ2	Change of department **R^2_{adj} = 14.6 %**							No change of department **R^2_{adj} = 18.3 %**						
	Regression coefficient	95% CI: minimum	95% CI: maximum	SE	β	T	P value	Regression coefficient	95% CI: minimum	95% CI: maximum	SE	β	T	P value
Konstante	4.356	3.758	4.954	0.305		14.294	≤ .001	3.947	3.736	4.157	0.107		36.768	≤ .001
Gender	0.043	-0.158	0.245	0.103	0.013	0.421	.673	0.160	0.086	0.233	0.038	0.049	4.233	≤ .001
Age (under/over 40)	-0.139	-0.329	0.051	0.097	-0.046	-1.435	.151	-0.163	-0.230	-0.095	0.035	-0.058	-4.716	≤ .001
Migration background	0.012	-0.238	0.262	0.127	0.003	0.096	.923	0.006	-0.091	0.104	0.050	0.001	0.128	.898
Hospital/ Other setting	-0.069	-0.259	0.121	0.097	-0.022	-0.711	.477	-0.003	-0.067	0.061	0.033	-0.001	-0.091	.927
Fulltime/ Part time	-.0208	-0.391	-0.024	0.093	-0.067	-2.221	.027	-0.073	-0.137	-0.008	0.033	-0.026	-2.209	.027
Contact with patients	0.139	-0.106	0.385	0.125	0.034	1.112	.266	0.064	-0.018	0.146	0.042	0.018	1.532	.126
Contact with infected patients	0.063	-0.191	0.318	0.130	0.021	0.488	.625	0.092	0.010	0.174	0.042	0.033	2.203	.028
Contact with contamined material	0.104	-0.141	0.348	0.125	0.034	0.830	.407	-0.061	-0.140	0.019	0.041	-0.022	-1.501	.133
Risk group age	-0.180	-0.474	0.113	0.149	-0.038	-1.208	.227	0.038	-0.052	0.127	0.046	0.010	0.824	.410
Risk group preexisting illness	0.021	-0.200	0.242	0.113	0.006	0.188	.851	0.150	0.073	0.227	0.039	0.043	3.805	≤ .001
Fear of becoming infected	0.131	0.043	0.218	0.044	0.107	2.938	.003	0.111	0.079	0.143	0.016	0.099	6.760	≤ .001
Fear of infecting family	0.117	0.035	0.199	0.042	0.103	2.801	.005	0.112	0.082	0.141	0.015	0.108	7.424	≤ .001
Social Support (ESSI)	-0.093	-0.116	-0.070	0.012	-0.240	-8.039	≤ .001	-0.082	-0.090	-0.074	0.004	-0.229	-19.705	≤ .001
Optimism	-0.209	-0.278	-0.141	0.035	-0.177	-5.972	≤ .001	-0.222	-0.246	-0.198	0.012	-0.210	-18.180	≤ .001

R^2_{adj} = adjusted R^2 (explained variance); SE = standard error; (manifestation coded by 0 vs. manifestation coded by 1); N = 7740

Supplement 4 Linear regression analysis for severity of generalized anxiety (GAD-2) symptoms for the subgroups depending on change of department

GAD2	Change of department **R^2_{adj} = 21.4 %**							No change of department **R^2_{adj} = 21.0 %**						
	Regression coefficient	95% CI: minimum	95% CI: maximum	SE	β	T	P value	Regressio n coefficien t	95% CI: minimum	95% CI: maximum	SE	β	T	P value
Konstante	3.766	3.187	4.345	0.295		12.767	≤ .001	3.451	3.233	3.668	0.111		31.074	≤ .001
Gender	0.134	-0.061	0.329	0.099	0.039	1.350	.177	0.234	0.158	0.310	0.039	0.069	6.001	≤ .001
Age (under/over 40)	0.136	-0.048	0.320	0.094	0.045	1.452	.147	0.036	-0.034	0.106	0.036	0.012	1.008	.313
Migration background	0.008	-0.234	0.250	0.123	0.002	0.065	.948	0.032	-0.069	0.133	0.052	0.007	0.624	.533
Hospital/ Other setting	0.019	-0.165	0.204	0.094	0.006	0.207	.836	0.151	0.085	0.218	0.034	0.051	4.479	≤ .001
Fulltime/ Part time	-0.043	-0.221	0.134	0.090	-0.014	-0.478	.633	0.013	-0.053	0.080	0.034	0.004	0.391	.696
Contact with patients	0.025	-0.213	0.263	0.121	0.006	0.205	.838	0.033	-0.052	0.118	0.043	0.009	0.768	.442
Contact with infected patients	0.295	0.049	0.541	0.125	0.097	2.350	.019	0.047	-0.038	0.132	0.043	0.016	1.089	.276
Contact with contamined material	-0.244	-0.481	-0.007	0.121	-0.080	-2.024	.043	-0.044	-0.126	0.039	0.042	-0.015	-1.038	.299
Risk group age	-.0160	-0.444	0.124	0.145	-0.033	-1.107	.268	-0.039	-0.131	0.054	0.047	-0.010	-.816	.415
Risk group preexisting illness	0.075	-0.139	0.288	0.109	0.020	0.685	.494	0.176	0.096	0.256	0.041	0.048	4.308	≤ .001
Fear of becoming infected	0.302	0.218	0.387	0.043	0.246	7.027	≤ .001	0.209	0.175	0.242	0.017	0.177	12.313	≤ .001
Fear of infecting family	0.094	0.015	0.174	0.040	0.082	2.334	.020	0.121	0.091	0.152	0.016	0.112	7.797	≤ .001
Social Support (ESSI)	-0.083	-0.105	-0.061	0.011	-0.213	-7.434	≤ .001	-0.081	-0.089	-0.072	0.004	-0.215	-18.807	≤ .001
Optimism	-0.245	-0.311	-0.178	0.034	-0.205	-7.220	≤ .001	-0.226	-0.251	-0.201	0.013	-0.203	-17.885	≤ .001

R^2_{adj} = adjusted R^2 (explained variance); SE = standard error; (manifestation coded by 0 vs. manifestation coded by 1); N = 7740

Supplement 5 Linear regression analysis for severity of depressive (PHQ-2) symptoms for the subgroups depending on direct contact with Covid-19 infected patients

PHQ2	Contact to COVID-19 infected patients R^2_{adj} =18.0 %							No contact to COVID-19 infected patients R^2_{adj} = 18.0 %						
	Regression coefficient	95% CI: minimum	95% CI: maximum	SE	β	T	P value	Regression coefficient	95% CI: minimum	95% CI: maximum	SE	β	T	P value
Konstante	4.103	3.779	4.428	0.165		24.816	≤ .001	4.365	4.083	4.647	0.144		30.373	≤ .001
Gender	0.138	0.034	0.243	0.053	0.045	2.597	.009	0.122	0.027	0.216	0.048	0.035	2.531	.011
Age (under/over 40)	-0.129	-0.233	-0.025	0.053	-0.046	-2.434	.015	-0.189	-0.270	-0.108	0.041	-0.067	-4.558	≤ .001
Migration background	0.016	-0.128	0.160	0.074	0.004	0.216	.829	0.001	-0.117	0.118	0.060	≤ 0.001	0.012	.990
Hospital/ Other setting	-0.066	-0.174	0.042	0.055	-0.020	-1.191	.234	0.021	-0.054	0.095	0.038	0.007	0.547	.584
Fulltime/ Part time	-0.056	-0.160	0.048	0.053	-0.018	-1.058	.290	-0.105	-0.181	-0.029	0.039	-0.038	-2.723	.006
Contact with patients	0.190	-0.005	0.384	0.099	0.032	1.913	.056	0.040	-0.046	0.127	0.044	0.012	0.910	.363
Change of department	-0.164	-0.288	-0.040	0.063	-0.043	-2.593	.010	-0.201	-0.311	-0.090	0.056	-0.047	-3.560	≤ .001
Contact with contamined material	0.028	-0.083	0.140	0.057	0.008	0.502	.616	-0.111	-0.217	-0.004	0.054	-0.028	-2.035	.042
Risk group age	-0.040	-0.181	0.101	0.072	-0.010	-0.560	.575	0.036	-0.073	0.145	0.056	0.009	0.651	.515
Risk group preexisting illness	0.067	-0.050	0.185	0.060	0.019	1.122	.262	0.172	0.079	0.266	0.048	0.049	3.606	≤ .001
Fear of becoming infected	0.120	0.074	0.166	0.023	0.109	5.145	≤ .001	0.106	0.065	0.146	0.021	0.089	5.129	≤ .001
Fear of infecting family	0.116	0.071	0.161	0.023	0.107	5.057	≤ .001	0.113	0.077	0.148	0.018	0.107	6.226	≤ .001
Social Support (ESSI)	-0.081	-0.093	-0.069	0.006	-0.226	-13.078	≤ .001	-0.085	-0.095	-0.075	0.005	-0.232	-16.759	≤ .001
Optimism	-0.229	-0.265	-0.193	0.018	-0.215	-12.498	≤ .001	-0.214	-0.243	-0.184	0.015	-0.198	-14.302	≤ .001

R^2_{adj} = adjusted R^2 (explained variance); SE = standard error; (manifestation coded by 0 vs. manifestation coded by 1); N = 7740

Supplement 6 Linear regression analysis for severity of generalized anxiety (GAD-2) symptoms for the subgroups depending on direct contact with COVID-19 infected patients

GAD2	Contact to COVID-19 infected patients **R^2_{adj} = 23.3 %**							No contact to COVID-19 infected patients **R^2_{adj} = 19.9 %**						
	Regression coefficient	95% CI: minimum	95% CI: maximum	SE	β	T	P value	Regression coefficient	95% CI: minimum	95% CI: maximum	SE	β	T	P value
Konstante	3.730	3.401	4.060	0.168		22.184	≤ .001	3.704	3.415	3.994	0.148		25.070	≤ .001
Gender	0.226	0.120	0.332	0.054	0.070	4.172	≤ .001	0.202	0.105	0.299	0.049	0.056	4.091	≤ .001
Age (under/over 40)	0.097	-0.009	0.202	0.054	0.033	1.793	.073	0.020	-0.064	0.104	0.043	0.007	.0471	.638
Migration background	-0.033	-0.180	0.114	0.075	-0.007	-0.441	.659	0.071	-0.050	0.191	0.062	0.015	1.147	.251
Hospital/ Other setting	0.077	-0.033	0.187	0.056	0.022	1.375	.169	0.169	0.092	0.245	0.039	0.058	4.330	≤ .001
Fulltime/ Part time	0.016	-0.090	0.122	0.054	0.005	0.294	.768	-0.004	-0.082	0.073	0.040	-0.001	-0.109	.913
Contact with patients	0.025	-0.172	0.223	0.101	0.004	0.250	.802	0.016	-0.073	0.105	0.045	0.005	0.349	.727
Change of department	-0.145	-0.271	-0.019	0.064	-0.036	-2.256	.024	-0.178	-0.292	-0.065	0.058	-0.040	-3.077	.002
Contact with contamined material	0.021	-0.092	0.134	0.058	0.006	0.364	.716	-0.159	-0.269	-0.049	0.056	-0.039	-2.845	.004
Risk group age	-0.081	-0.225	0.062	0.073	-0.019	-1.113	.266	-0.040	-0.152	0.073	0.057	-0.010	-0.692	.489
Risk group preexisting illness	0.139	0.019	0.258	0.061	0.037	2.271	.023	0.177	0.081	0.273	0.049	0.049	3.605	≤ .001
Fear of becoming infected	0.235	0.189	0.282	0.024	0.203	9.928	≤ .001	0.210	0.169	0.252	0.021	0.171	9.937	≤ .001
Fear of infecting family	0.123	0.078	0.169	0.023	0.108	5.298	≤ .001	0.117	0.081	0.154	0.019	0.107	6.298	≤ .001
Social Support (ESSI)	-0.089	-0.101	-0.077	0.006	-0.236	-14.136	≤ .001	-0.075	-0.086	-0.065	0.005	-0.198	-14.436	≤ .001
Optimism	-0.231	-0.268	-0.195	0.019	-0.206	-12.404	≤ .001	-0.226	-0.256	-0.196	0.015	-0.201	-14.717	≤ .001

R^2_{adj} = adjusted R^2 (explained variance); SE = standard error; (manifestation coded by 0 vs. manifestation coded by 1); N = 7740

Supplement 7 Linear regression analysis for severity of depressive (PHQ-2) symptoms for the subgroups depending on the presence of a preexisting illness

PHQ2	Risk group because of preexisting illness R^2_{adj} = 20.1 %							No Risk group because of preexisting illness R^2_{adj} = 16.7 %						
	Regression coefficient	95% CI: minimum	95% CI: maximum	SE	β	T	P value	Regression coefficient	95% CI: minimum	95% CI: maximum	SE	β	T	P value
Konstante	4.239	3.759	4.720	0.245		17.307	≤ .001	4.140	3.909	4.371	0.118		35.169	≤ .001
Gender	0.061	-0.106	0.228	0.085	0.017	0.717	.473	0.156	0.080	0.232	0.039	0.049	4.023	≤ .001
Age (under/over 40)	-0.064	-0.231	0.103	0.085	-0.019	-0.748	.454	-0.179	-0.248	-0.111	0.035	-0.066	-5.107	≤ .001
Migration background	0.064	-0.164	0.291	0.116	0.013	0.549	.583	-0.002	-0.101	0.097	0.050	≤ 0.001	-0.041	.967
Hospital/ Other setting	0.022	-0.124	0.168	0.074	0.007	0.299	.765	-0.021	-0.088	0.046	0.034	-0.007	-0.610	.542
Fulltime/ Part time	-0.074	-0.221	0.073	0.075	-0.024	-0.992	.321	-0.093	-0.160	-0.026	0.034	-0.033	-2.727	.006
Contact with patients	-0.016	-0.199	0.168	0.094	-0.004	-0.167	.868	0.101	0.015	0.187	0.044	0.028	2.301	.021
Change of department	-0.080	-0.277	0.118	0.101	-0.018	-0.794	.427	-0.207	-0.298	-0.117	0.046	-0.052	-4.489	≤ .001
Contact with infected patients	0.159	-0.027	0.344	0.094	0.052	1.680	.093	0.068	-0.019	0.154	0.044	0.024	1.530	.126
Contact with contamined material	0.014	-0.166	0.195	0.092	0.005	0.154	.878	-0.047	-0.130	0.037	0.043	-0.017	-1.100	.271
Risk group age	-0.012	-0.180	0.157	0.086	-0.003	-0.136	.892	0.012	-0.089	0.114	0.052	0.003	0.235	.814
Fear of becoming infected	0.155	0.086	0.223	0.035	0.132	4.437	≤ .001	0.100	0.067	0.134	0.017	0.087	5.842	≤ .001
Fear of infecting family	0.137	0.070	0.203	0.034	0.122	4.040	≤ .001	0.108	0.077	0.138	0.016	0.105	6.914	≤ .001
Social Support (ESSI)	-0.098	-0.115	-0.081	0.009	-0.273	-11.268	≤ .001	-0.079	-0.087	-0.070	0.004	-0.217	-17.983	≤ .001
Optimism	-0.214	-0.268	-0.160	0.027	-0.190	-7.826	≤ .001	-0.221	-0.246	-0.196	0.013	-0.208	-17.309	≤ .001

R^2_{adj} = adjusted R^2 (explained variance); SE = standard error; (manifestation coded by 0 vs. manifestation coded by 1); N = 7740

Supplement 8 Linear regression analysis for severity of generalized anxiety (GAD-2) symptoms for the subgroups depending on the presence of a preexisting illness

GAD2	Risk group because of preexisting illness **R^2_{adj} = 23.0 %**							No Risk group because of preexisting illness **R^2_{adj} = 19.3 %**						
	Regression coefficient	95% CI: minimum	95% CI: maximum	SE	β	T	P value	Regression coefficient	95% CI: minimum	95% CI: maximum	SE	β	T	P value
Konstante	3.825	3.327	4.324	0.254		15.052	≤ .001	3.593	3.358	3.829	0.120		29.903	≤ .001
Gender	0.140	-0.033	0.313	0.088	0.038	1.590	.112	0.239	0.161	0.317	0.040	0.072	6.030	≤ .001
Age (under/over 40)	0.060	-0.113	0.234	0.088	0.017	0.685	.494	0.049	-0.021	0.119	0.036	0.017	1.369	.171
Migration background	-0.004	-0.239	0.232	0.120	-0.001	-0.029	.977	0.041	-0.060	0.142	0.051	0.009	0.788	.431
Hospital/ Other setting	0.117	-0.034	0.269	0.077	0.036	1.519	.129	0.141	0.072	0.209	0.035	0.048	4.039	≤ .001
Fulltime/ Part time	-0.014	-0.166	0.139	0.078	-0.004	-.177	.860	0.007	-0.061	0.076	0.035	0.003	0.212	.832
Contact with patients	0.043	-0.147	0.234	0.097	0.011	0.445	.656	0.027	-0.060	0.115	0.045	0.007	0.612	.541
Change of department	-0.109	-0.314	0.095	0.104	-0.024	-1.048	.295	-0.171	-0.263	-0.078	0.047	-0.042	-3.626	≤ .001
Contact with infected patients	0.126	-0.066	0.319	0.098	0.039	1.290	.197	0.056	-0.033	0.144	0.045	0.019	1.230	.219
Contact with contamined material	-0.104	-0.291	0.084	0.095	-0.032	-1.085	.278	-0.052	-0.137	0.033	0.043	-0.018	-1.192	.233
Risk group age	-0.026	-0.201	0.148	0.089	-0.007	-0.297	.767	-0.064	-0.167	0.040	0.053	-0.015	-1.208	.227
Fear of becoming infected	0.253	0.182	0.324	0.036	0.205	6.997	≤ .001	0.211	0.177	0.246	0.018	0.178	12.069	≤ .001
Fear of infecting family	0.156	0.087	0.224	0.035	0.131	4.438	≤ .001	0.110	0.079	0.141	0.016	0.103	6.909	≤ .001
Social Support (ESSI)	-0.092	-0.110	-0.074	0.009	-0.241	-10.152	≤ .001	-0.078	-0.087	-0.069	0.004	-0.206	-17.360	≤ .001
Optimism	-0.222	-0.278	-0.167	0.028	-0.187	-7.845	≤ .001	-0.229	-0.255	-0.204	0.013	-0.209	-17.623	≤ .001

R^2_{adj} = adjusted R^2 (explained variance); SE = standard error; (manifestation coded by 0 vs. manifestation coded by 1); N = 7740

Supplement 9. Independent variables (IV) for severity of depressive and generalized anxiety symptoms in the subgroups men, women, department changers, non-department changers, those with/without direct contact to COVID-19 patients and those with/without a preexisting illness.

IV	PHQ-2	GAD-2
**Social support**	Significant IV in all subgroups (β = -0.217 – (-0.273)); most important predictor in all subgroups except in men	Significant IV in all subgroups (β = - 0.198 – (-0.241); most important predictor in all but men, department changers and those without direct contact to infected patients
**Optimism**	Significant IV for all groups (β = -0.177 – (-0.230)); most important factor for men	Significant IV in all subgroups (β = - 0.187 – (-0.225)); most important factor in men and those without direct contact with infected patients
**Fear of becoming infected**	Significant IV for all subgroups (β = 0.102-0.132 for women, department changers, those with a preexisting illness; β < 0.099 for all others)	Significant IV in all groups (β = 0.171-0.205); most important factor in department-changers
**Fear of infecting relatives**	Significant IV for all subgroups (β = 0.097 for women; β = 0.103-0.143 for all others)	Significant IV in all subgroups (β = 0.082 for department changers; β = 0.103-0.121 for all others)
**Gender**	Significant IV for all but department changers and those with a preexisting illness (β = 0.035-0.049)	Significant IV in all groups except for department changers and those with a preexisting illness (β = 0.056-0.072)
**Change of department**	Significant IV in all subgroups but those with a preexisting illness (β = -0.035-(-0.077))	Significant IV in all subgroups except in those with a preexisting illness (β = -0.031- (-0.061)
**Direct contact with infected patients**	Significant IV only for non-department-changers (β = 0.033) (direct contact was associated to less depressive symptoms)	Significant IV only for department changers (β = 0.097) (direct contact was associated to less anxiety symptoms)
**Preexisting illness**	Significant IV for all except department changers and those with contact with infected patients (β = 0.035-0.049)	Significant IV for all groups except department changers (β = 0.037-0.066)

$p < 0.05$ for all reported β values
